# 14-day pantoprazole- and amoxicillin-containing high-dose dual therapy for *Helicobacter pylori* eradication in elderly patients: A prospective, randomized controlled trial

**DOI:** 10.3389/fphar.2023.1096103

**Published:** 2023-02-01

**Authors:** Qinyu Yang, Cong He, Yi Hu, Junbo Hong, Zhenhua Zhu, Yong Xie, Xu Shu, Nonghua Lu, Yin Zhu

**Affiliations:** Department of Gastroenterology, Digestive Disease Hospital, The First Affiliated Hospital of Nanchang University, Nanchang, China

**Keywords:** bismuth, dual therapy, elderly patients, eradication, *Helicobacter pylori*

## Abstract

**Background:** Currently, the management of *Helicobacter pylori* (*H. pylo*ri) infection in elderly patients is controversial. We investigated whether high-dose dual therapy would serve as the first-line therapy in elderly patients.

**Methods:** This was a single-center, randomized study of 150 elderly patients with *H. pylori* infection who were randomly assigned to 14-day therapy with pantoprazole 40 mg 3 times daily and either amoxicillin 1,000 mg 3 times daily or amoxicillin 1,000 mg twice daily, clarithromycin 500 mg twice daily and bismuth 220 mg twice daily. *H. pylori* eradication was evaluated by a 13C-urea breath test 4 weeks after the completion of treatment.

**Results:** Successful eradication was achieved in 89.3% of the high-dose dual therapy (HT) group in the intention‐to‐treat (ITT) analysis, 91.7% in the modified intention-to-treat (mITT) analysis, and 93.0% for per‐protocol (PP) analysis which was similar to the bismuth-containing quadruple therapy (BQT) group (86.6%, 87.8%, and 90.3%, respectively). There were no significant difference between the HT group and the BQT group in the ITT analysis (*p* = 0.484), mITT analysis (*p* = 0.458), or PP analysis (*p* = 0.403). HT was associated with fewer side effects (10.6% of patients) than BQT (26.6%) (*p* = 0.026).

**Conclusion:** In this trial, we found that 14-day HT had a similar eradication rate to BQT but fewer side effects, which may be better for elderly patients.

## 1 Introduction

*Helicobacter pylori* (*H. pylori*) infection and related diseases are a major global health concern. Approximately 44% of the global population is infected with *H. pylori* (Malfertheiner et al., 2017). The prevalence of upper gastrointestinal diseases is increasing in elderly populations worldwide. Approximately 53%–73% of elderly peptic ulcer patients are infected with *H. pylori*; however, elderly *H. pylori*-positive patients are less likely to receive treatment (Pilotto, 2004). Peptic ulcers are currently the main impact of *H. pylori* on healthcare resources, and older patients are at the greatest risk, as the mortality rate from peptic ulcers increases from approximately 1 in 100 000 at age 20 to 2-3 in 100 000 at age 70 (Bank et al., 1986). The Fifth Chinese National Consensus Report on management highly recommends that patients with peptic ulcers receive eradication of *H. pylori* (Liu et al., 2018). A study conducted by Leung et al. (Leung et al., 2018) demonstrated that among patients infected with *H. pylori* who received eradication therapy, the gastric cancer risk was significantly lower in the elderly compared with the matched general population highlighting the importance of eradication therapy for elderly patients.

A proton pump inhibitor (PPI) and the antibiotics clarithromycin and amoxicillin/metronidazole, the so-called standard triple therapy, are used as the first-line eradication therapy for *H. pylori* infection, worldwide (Salles and Mégraud, 2007). However, this kind of treatment may cause resistance-associated *H. pylori* eradication failure due to increasing antimicrobial resistance. Bismuth-containing quadruple therapy (BQT) is commonly used in China and is superior to the standard triple therapy (Xie et al., 2018). In general, elderly patients show a low tolerance and compliance to *H. pylori* eradication therapy (Salles and Mégraud, 2007). In some countries and regions, it is difficult to obtain drugs such as bismuth and tetracycline. Moreover, the risk of adverse effects of *H. pylori* eradication therapy increases in elderly patients. Therefore, comprehensive benefit and risk assessment for elderly patients undergoing *H. pylori* eradication treatment are essential.

In the 1990s, some European scholars proposed a dual therapy regimen of PPI plus an antibiotic, but compared with the triple regimen proposed in early guidelines, this regimen has a lower eradication rate, so it is not widely used in clinical treatment (Unge et al., 1989). However, according to some recent literature reports, increasing the dose of PPI and/or amoxicillin in the dual therapy regimen can effectively eradicate *H. pylori* (Goh et al., 2012; Attumi and Graham, 2014; Yang et al., 2015; Yu et al., 2019). Some studies have shown that the drug resistance of the bismuth quadruple regimen after eradication failure is significantly higher than that of the dual therapy (Ren et al., 2014). In China, the rural population accounts for the main proportion of patients with *H. pylori* infection. In regard to elderly patients, the instructions for taking two drugs are more concise and easier to understand than the instructions for taking four drugs. Several meta-analyses have compared the efficacy and safety of dual therapy and other regimens, and all of them concluded that PPI-amoxicillin is equally effective as other commonly used therapeutic regimens in the eradication of *H. pylori* (Gao et al., 2020a; Li et al., 2021). Therefore, high-dose dual therapy (HT) may be an effective eradication regimen for elderly patients who are not allergic to penicillin.

The widespread presence of *H. pylori* in elderly patients and the role of *H. pylori* in peptic ulcers, precancerous lesions of gastric cancer, gastric cancer, and gastric lymphoma (MALT) make a diagnosis and eradication therapy particularly important in this population. However, there is currently no consensus or guideline on *H. pylori* eradication therapy in elderly patients. Therefore, it is of practical significance to develop a safe and effective *H. pylori* eradication program for elderly patients, which is worthy of further study.

## 2 Materials and methods

### 2.1 Subjects and study design

This single-center, prospective, open-label, randomized controlled trail was conducted at the First Affiliated Hospital of Nanchang University, Nanchang, China. Between February 2020 and February 2022, a total of 162 eligible cases were planned, 8 patients met the exclusion criteria, 4 patients refused to participate, and 150 patients were enrolled in outpatient clinics. Our study followed the recommendations of the Consolidated Standards of Reporting Trials (CONSORT) statement for reporting randomized controlled trials. Written informed consent was obtained from all participants. The study protocol was approved by the Institutional Ethics Board of the First Affiliated Hospital of Nanchang University, Nanchang, China (No. 101.2019). The trial was registered in the Chinese Clinical Trials Registration (chictr.org.cn), registered number is ChiCTR2000029679.

Detailed inclusion criteria were as follows: 1). male or female patients aged ≥60 years; 2) patients diagnosed with *H*. *pylori* infection by positive C-urea breath test (UBT) or by positive gastric mucosa biopsy results (UBT results between 4‰ and 6‰ were not included in the group, as well as those who remained above 4‰ after 7 days of reexamination); 3). patients who signed the informed consent form.

The exclusion criteria were as follows: 1). patients who had received standard *H. pylori* eradication therapy before enrollment; 2). patients who had taken antibiotics, bismuth, and PPIs within 4 weeks before initiating treatment; 3). alcohol abuse (daily alcohol intake exceeding 50 G for more than 6 months); 4). patients with a serious primary diseases of the liver, kidney, heart, brain, lung, endocrine system, and hematopoietic system; 5). clinically significant liver or kidney insufficiency (transaminase greater than 1.5 times the upper limit of normal, or serum creatinine greater than the upper limit of normal; 6). patients who were allergic to drugs related to medications.

### 2.2 Treatment regiments

The HT consisted of pantoprazole 40 mg 3 times daily and amoxicillin 1,000 mg 3 times daily for 14 days. Patients were advised to take pantoprazole 30 min before breakfast, lunch, and dinner, and amoxicillin after breakfast, lunch, and dinner.

The BQT consisted of pantoprazole 40 mg twice daily, amoxicillin 1,000 mg twice daily, clarithromycin 500 mg twice daily, and bismuth 220 mg twice daily for 14 days. Pantoprazole and bismuth were taken 30 min before breakfast and dinner, and amoxicillin and clarithromycin were taken 30 min after breakfast and dinner.

### 2.3 Determination of successful eradication

Negative UBT results 4–6 weeks after the end of treatment indicated successful *H*. *pylori* eradication. Side effects and compliance were assessed using a questionnaire administered after the end of therapy. Compliance was defined as good when more than 80% of the total pills were taken. Side effects were graded according to their influence on daily life, classified as “mild” (transient and well-tolerated), “moderate” (discomfort that partially affected daily life), or “severe” (severe interruption of daily activities).

### 2.4 Sample size estimation and statistical analysis

The purpose of this study was verify whether HT could achieve a similar effective eradication rate to BQT in elderly patients. Based on a previous study, we estimated the eradication rate of the BQT groups to be 89.7% (Yang et al., 2019a). We assumed an eradication rate of 90% in both the BQT and HT groups; with a non-inferiority margin delta (δ) = 0.1 (10%), *α* = 0.05 (two-sided), and 1-β = 0.90, we expected to recruit at least 75 patients per group (assuming a 10% loss to follow-up).

Statistical analyses to identify prediction factors were performed using SPSS 25.0 for Windows (SPSS, Chicago, IL). Continuous variables are expressed as the mean ± standard deviation, and categorical data are presented as absolute numbers and percentages. The chi-square test was used to determine the significance of differences between categorical variables and the *t*-test were used for continuous variables. The eradication rate of *H. pylori* was calculated using intention-to-treat (ITT), modified intention-to-treat (mITT), and per-protocol (PP) analyses together with a 95% confidence interval (CI).

## 3 Results

### 3.1 Basic patient demographics

**FIGURE 1:**
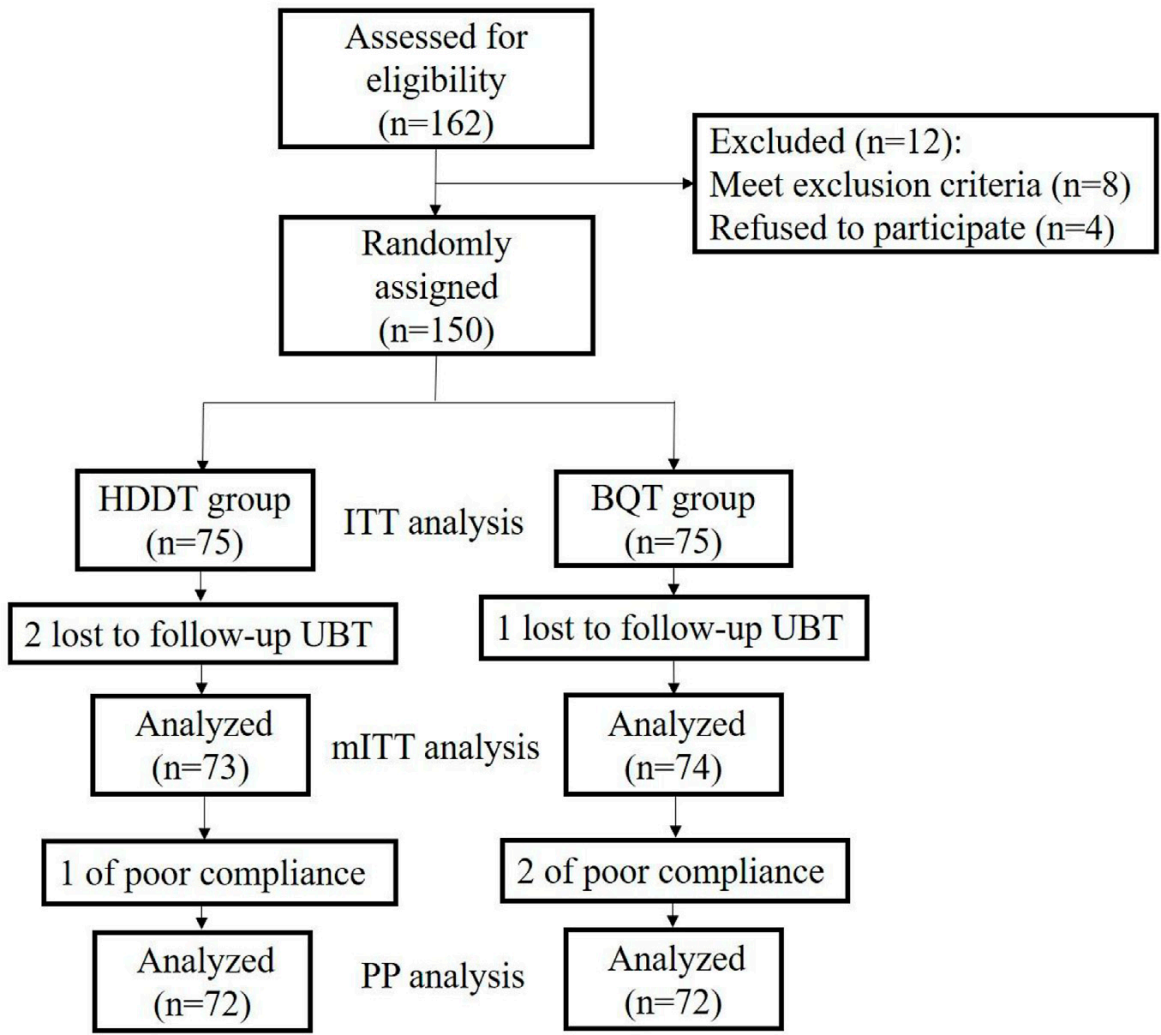
Flow diagram of this study. HT, high‐dose dual therapy, BQT, bismuth quadruple therapy, ITT, intension-to-treat, PP, pre-protocol.

A total of 162 patients were assessed for eligibility, and 150 patients were recruited for this study. In the HT group, 2 patients were lost to follow-up, and 1 paytient was not compliant with medications. In the BQT group, 1 patient was lost to follow-up, and 2 patients were not compliant with medications (Figure 1).

The distribution of patients according to sex, symptoms, and mean age is shown in Table 1. There were no significant difference between the two groups regarding clinical characteristics.

**TABLE 1:** Demographic and clinical data of patients.

Variables	HT group	BQT group	*p*-value
Total	75 (50.0)	75 (50.0)	—
Age (mean, SD), y	67.11 ± 5.2	66.67 ± 5.33	0.625
Sex			0.339
Male	37 (49.3)	33 (44.0)	
Female	48 (50.7)	42 (56.0)	
Hypertension	7 (9.3)	5 (6.6)	0.858
Diabetes	4 (5.3)	3 (4.0)	0.774
Smoking	8 (10.6)	7 (9.3)	0.569
Drinking	3 (4.0)	4 (5.3)	0.974
Symptom			
Bloating	8 (10.6)	5 (6.6)	0.765
Belching	6 (8.0)	9 (12.0)	0.577
Ozostomia	3 (4.0)	3 (4.0)	1.0
Abdominal pain	10 (13.3)	12 (16.0)	0.583
Diarrhea	6 (8.0)	6 (8.0)	1.0
None symptom	42 (56.1)	40 (53.4)	0.661
Loss of follow-up	2	1	0.946

Abbreviations: HT, high‐dose dual therapy; BQT, bismuth quadruple therapy.

### 3.2 *H. pylori* eradication rates

In the ITT analysis, *H. pylori* was eradicated in 89.3% of patients (67/75) (95% Cl: 82.2–96.5) in the HT group compared with 86.6% (65/75) (95% Cl: 78.8–94.5) in the BQT group (Table 2). In the mITT analysis, *H. pylori* were eradicated in 91.7% of patients (67/73) (95% Cl:85.3–98.2) in the HT group compared with 87.8% (65/74) (95% Cl:80.2–95.5) in the BQT group (Table 2). In the PP analysis, *H. pylori* was eradicated in 93.0% of patients (67/72) (95% Cl: 87.0–99.1) in the HT group compared with 90.3% (65/72) (95% Cl: 83.3–97.3) in the BQT group (Table 2).

**TABLE 2:** Eradication rates in each regimen.

Variables	HT group	BQT group	*p*-value
Total	75	75	
ITT	67/75 (89.3)	65/75 (86.6)	0.484
95% Cl	82.2–96.5	78.8–94.5	
mITT	67/73 (91.7)	65/74 (87.8)	0.458
95% Cl	85.3–98.2	80.2–95.5	
PP	67/72 (93.0)	65/72 (90.3)	0.403
95% Cl	87.0–99.1	83.3–97.3	

Abbreviations: HT, high‐dose dual therapy; BQT, bismuth quadruple therapy; CI, confidence interval; ITT, intention‐to‐treat; mITT, modified intention‐to‐treat; PP, per‐protocol.

There were no significant difference in the eradication rates in the ITT, mITT, and PP analysis between the 2 treatment groups.

### 3.3 Side effects and compliance

Side effects included nausea, vomiting, bloating, abdominal pain, diarrhea, and skin rash (Table 3). In the HT group, the frequency of side effects was 10.6% (8/75) of patients. In the BQT group, the frequency of side effects was 26.6% (20/75) of patients (*p* = 0.026). The side effects were all mild and moderate. Treatment was reported as tolerable except for one patient in the HT group and two patients in the BQT group who could not tolerate the treatment regimen due to skin rash or nausea. The compliance rates was 98.7% (74/75) in the HT group and 97.3% (73/75) in the BQT group.

**TABLE 3:** Variety of adverse events in each regimen.

Variables	HT group	BQT group	*p*-value
Total	8 (10.6)	20 (26.6)	0.026
Adverse events grades			
Mild	6	16	
Moderate	2	4	
Severe	0	0	
Adverse events			
Nausea	4	9	
Vomiting	1	2	
Abdominal pain	1	1	
Bloating	0	2	
Diarrhea	2	6	
Skin rash	0	0	
Poor compliance	1	2	0.977

Abbreviations: HT, high‐dose dual therapy; BQT, bismuth quadruple therapy.

### 3.4 Previous literature review

A previous literature review is illustrated in Table 4, which includes. 9 studies conducted in China and published in the last 5 years. Rabeprazole and esomeprazole were the most commonly used PPIs in these studies. Most studies reported a low incidence of adverse reactions to PPI-amoxicillin dual therapy.

**TABLE 4:** Characteristics of studies.

Studies	Subgroup	Regiments	Eradication rate (ITT/PP, %)	Adverse events rate (%)	Compliance (%)
Yang et al., 2019a	DT (116) Control (116)	E 20 mg qid + A 750 mg qid*14 d E 20 mg bid + B 1 g bid + A 1 g bid + C 500 mg bid*14 d	87.9/91.1 89.7/91.2	6.3 22.8	96.6 98.3
Song et al., 2020	DT (380) Control (380)	E 20 mg qid + A 750 mg qid*14 d E 20 mg bid + B 220 mg bid + A 1 g bid + C 500 mg bid*14 d	87.1/92.4 80.5/87.8	17.6 25.5	96.3 92.3
Gao et al., 2020b)	DT (198)	R 10 mg tid + A 1000 mg tid*14 d	90.9	11.1	—
Yu et al., 2019	DT (80) Control (80)	E 40 mg bid + A 1000 mg tid*14 d E 40 mg bid + A 1000 mg tid + B 220 mg bid *14 d	88.8/93.3 92.5/96.1	11.3 8.8	98.7 97.5
Zhang et al., 2020	DT (104) Control (104)	E 20 mg qid + A 750 mg qid*10 d E 20 mg qid + A 1000 mg tid*14 d	79.8/81.3 83.5/86.4	5.9 5.0	97.1 97.1
Hu et al., 2017	DT (87) DT (87) Control (89)	R10 mg qid + A 750 mg qid*14 d R 20 mg qid + A 750 mg qid*14 d E 20 mg bid + B 220 mg bid + A 1 g bid + C 500 mg bid*14 d	78.1/79.1 81.6/83.5 84.3/86.2	3.4 5.7 11.2	98.9 97.7 97.8
Shen et al., 2022	DT (496) Control (475)	E 20 mg qid + A 750 mg qid*14 d E 20 mg bid + B 220 mg bid + A 1 g bid + C 250 mg bid*14 d	88.3/91.6 85.2/90.6	13.3 28.2	99.2 99.2
Zou et al., 2021	DT (104) Control (116)	E 20 mg qid + A 750 mg qid*10 d E 20 mg qid + A 750 mg qid*14 d	78.4/80.0 89.7/92.9	6.8 5.7	80.0 92.9
Tai et al., 2019	DT (115) Control (114)	E 40 mg qid + A 750 mg qid*14 d E 40 mg bid + M 500 mg bid + A 1 g bid + C 250 mg bid*14 d	91.7/95.7 86.7/92.0	9.6 23.0	100 100

Abbreviations: A, amoxicillin; B, bismuth; C, clarithromycin; DT, dual therapy; E, esomeprazole; ITT, intention-to-treat analysis; L, levofloxacin; M, metronidazole; O, omeprazole; PP, per-protocol analysis; R, rabeprazole; T, tetracycline.

## 4 Discussion

In the current study, we conducted the first, randomized, controlled trial to comparing the efficacy of 14-day hybrid and 14-day bismuth quadruple therapies for the first-line treatment of *H. pylori* infection in elderly patients. Although dual therapy is not currently recommended as the first-line regimen for treating *H pylori* infection, many studies have reported an effective eradication rate of this regimen (Ren et al., 2014; Yang et al., 2015; Tai et al., 2019). Our results showed that both regimens achieved a successful eradication rate, and HT with pantoprazole and amoxicillin was efficient with a rate of 93.0% in the PP analysis, 91.7% in the mITT analysis and 89.3% in the ITT analysis. Side effects were few although a minority of patients in the BQT group complained of vomiting due to clarithromycin. The HT was generally well tolerated by patients.

Currently, there are no specific guidelines regarding the management of *H. pylori* infection in elderly patients. With the increased antibiotic resistance of *H. pylori*, due to previous multiple exposures to antibiotics, elderly patients fail their intended eradication therapy. Moreover, *H. pylori* microbiology summarizes the existing evidence for first- and second-line treatment regimens that may be considered for special populations such as the elderly. Elderly patients generally have renal or liver dysfunction, which might affect drug accumulation. As BQT contains more antibiotics than HT, patients tend to experience more side effects. Individual treatment needs to be performed in elderly patients.

The globally increasing antibiotic resistance in recent years is an important cause of first-line *H pylori* eradication failure (Graham and Fischbach, 2010). To overcome this problem, there are several novel therapies such as sequential therapy, non-bismuth quadruple therapy, and hybrid therapy that have achieved high eradication rates in first-line *H pylori* eradication (Liou et al., 2013; Liou et al., 2016). Recently, dual therapy has gained attention as both first-line and rescue therapy. However, studies from the different regions show different cure rates, probably due to different dosages of PPI and amoxicillin (Graham et al., 2010; Kwack et al., 2016; Park et al., 2017). Some comments on the heterogeneity of the results with HT should be included. In this respect, some meta-analyses showed higher HT effectiveness in studies in Asian countries than in European countries (Yang et al., 2019b; Gao et al., 2020a; Zhu et al., 2020). There have also been several dual-therapy studies conducted in China, and most of them show successful eradication rates with fewer adverse events (Hu et al., 2017; Tai et al., 2019; Gao et al., 2020b; Song et al., 2020; Zhang et al., 2020; Zou et al., 2021; Shen et al., 2022) (Table 4). Therefore, we questioned whether high-dose and high-frequency amoxicillin-PPI dual therapy could be widely used in elderly patients.

Amoxicillin is widely used in the treatment of *H pylori* infections; it is one of the most active antimicrobials against *H pylori in vivo* with a minimum inhibitory concentration of ≤0.01–0.1 mg/L (Hirschl and Rotter, 1996). An improvement in acid inhibition extending into the nighttime hours, maintaining an intragastric pH of 6 or above, would greatly improve the eradication rates of a regiment (Marcus et al., 2012). Several studies have tested the effect of a total daily dose of amoxicillin on the cure rate. There is evidence that a dose of 3 g per day can achieve an effective eradication rate (Kwack et al., 2016; Li et al., 2021).

Sufficient acid inhibition is necessary because intragastric pH is one of the critical determinants and amoxicillin is acid labile. In aition, other factors influence the efficacy of treating *H. pylori* infection. The PPI potency has a strong ability to reliably maintain a relatively high intragastric pH. A previous study evaluating the efficacy of different PPIs combined with bismuth quadruple regimens for *H. pylori* eradication demonstrated that the eradication rates between different PPI regimens were similar in treating *H. pylori* infection (Kan et al., 2020). To achieve a reliable cure rate, a high dose of PPI that is unaffected by CYP2C19 genotypes may be needed. The pantoprazole-based eradication program was less affected by the *CYP2C19* polymorphism (Fu et al., 2021). In this study, we used 40 mg pantoprazole which is relatively unaffected by CYP2C19 metabolism. A study conducted by Zullo et al. (2015) showed that a 10-day HT (esomeprazole 40 mg three times daily and amoxicillin 1 g three times daily) was effective and safe as a first-line treatment for *H. pylori* infection in Italy. However, the eradication rate was below 90%. Ping et al. (Hsu et al., 2018) found that *H pylori* infection was eradicated in 96.6% of patients who received reverse hybrid therapy (pantoprazole 40 mg plus amoxicillin 1 g twice daily for 14 days, and clarithromycin 500 mg plus metronidazole 500 mg twice daily for the first 7 days). Therefore, the regimen of 40 mg pantoprazole plus 3 g amoxicillin per day may be a favorable choice for elderly patients.

In the HT group, there were 11 patients who concomitantly and continually took medications, such as statins, hypotension, hypoglycemics, and antiplatelet drugs. Successful eradication was achieved in 10 of these patients. Thus, a dual regimen with fewer antibiotics would be an option for older patients or those with multiple comorbidities. Adverse events occurred in 10.6% (8/75) of patients, which was similar to most reports (Tai et al., 2019; Zou et al., 2021). Nausea, diarrhea, and abdominal pain were the most frequently occurring adverse events. All adverse events disappeared spontaneously after treatment.

Our study had some limitations. First, we did not perform susceptibility testing which was directly correlated. Second, we did not measure the patients’ intragastric pH value or CYP2C19 genotype. This was also a single-center study. A multicenter study should be conducted to confirm our findings.

In conclusion, our data showed that a 14-day regimen with pantoprazole 40 mg 3 times daily and amoxicillin 1,000 mg 3 times daily achieved an *H. pylori* eradication rate of 89.3%. This HT had few side effects and avoided the prevalence of antibiotic resistance. Clinicians can choose this treatment when considering *H. pylori* eradication in elderly patients.

## Data Availability

The original contributions presented in the study are included in the article/supplementary material, further inquiries can be directed to the corresponding authors.
